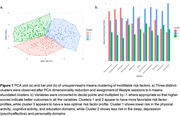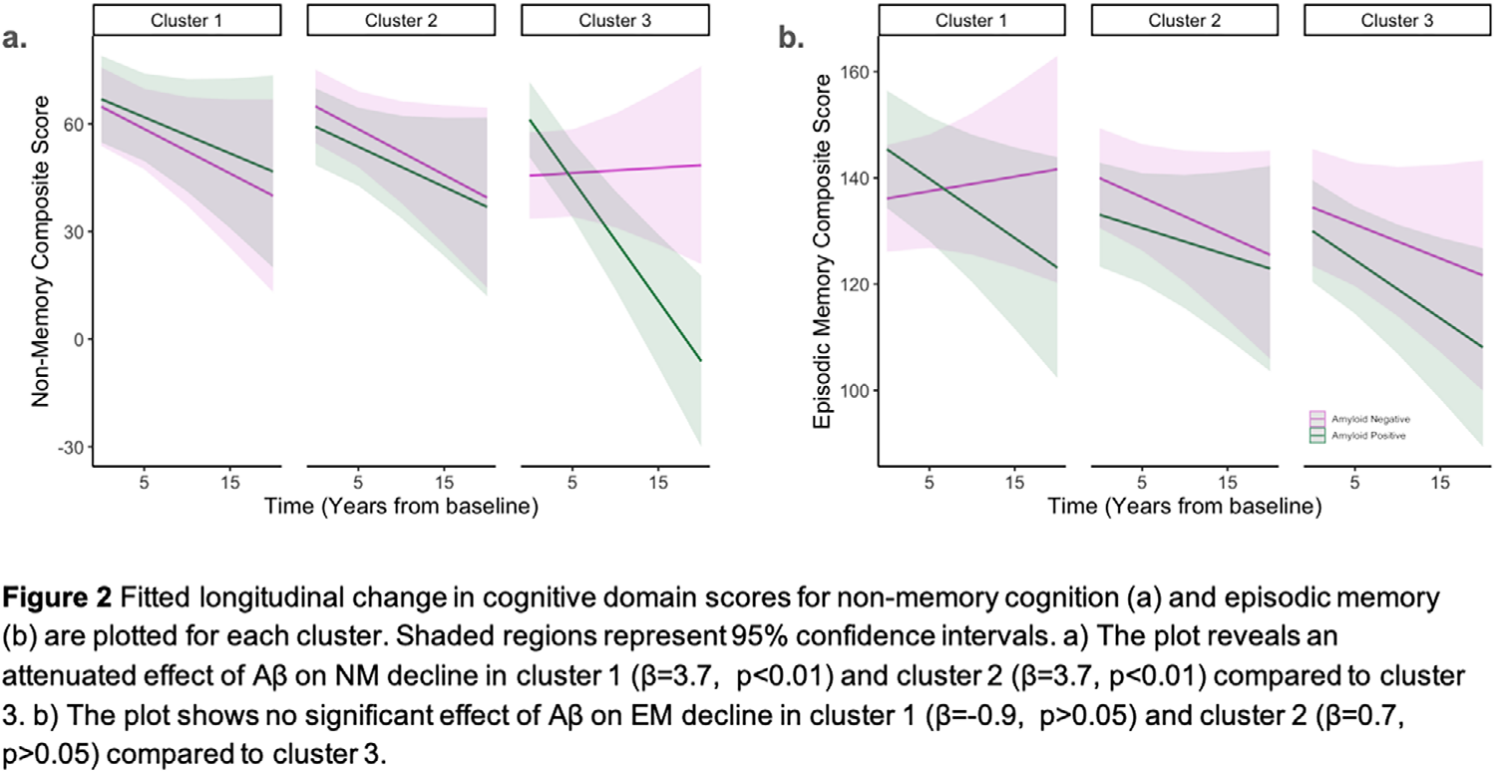# Clusters of modifiable risk factors provide resilience to the cognitive effects of β‐amyloid pathology in aging

**DOI:** 10.1002/alz.093917

**Published:** 2025-01-09

**Authors:** Mohini Bhade, Stefania Pezzoli, Joseph Giorgio, Tyler J. Ward, Susan M. Landau, William J. Jagust

**Affiliations:** ^1^ University of California, Berkeley, Berkeley, CA USA; ^2^ Lawrence Berkeley National Laboratory, Berkeley, CA USA

## Abstract

**Background:**

Although it has been estimated that modifiable risk factors account for around 40% of population variability in dementia risk, understanding how risk factors are related to one another and to brain pathology and cognition has been challenging. We used a clustering approach to examine patterns of risk factor interrelationships and to investigate how these patterns affect relationships between pathology and cognition.

**Method:**

We collected risk factor data concerning health, lifestyle, sleep, and personality from 149 cognitively normal older adults (73±6.2 years), as well as PET‐measured amyloid (11C‐Pittsburgh compound‐B, PiB) and, in a subsample (n = 126), PET‐measured tau (18F‐Flortaucipir, FTP). All participants completed longitudinal neuropsychological testing (follow‐up 8.1±4.2 years). K‐means clustering was used to assign participants to different risk profiles that reflected how risk factors were interrelated. Linear mixed‐effects models were used to assess associations between decline in 1) episodic memory (EM) and 2) non‐memory (NM) cognition composite scores, with cluster assignment and PiB‐status (±) or entorhinal FTP uptake as predictors. Analyses were age and sex adjusted.

**Result:**

K‐means clustering generated 3 distinct risk‐related profiles, with more favorable profiles seen for participants in cluster 1 (physical/cognitive activity, education) and cluster 2 (sleep, depression, personality) than cluster 3 (Figure 1). In a model predicting NM decline with PiB‐status as a predictor, we observed a significant three‐way interaction between PiB‐status, cluster, and time (p<0.01) with an attenuated effect of Aß on NM decline in cluster 1 (ß = 3.7, p<0.01) and cluster 2 (ß = 3.7, p<0.01) compared to cluster 3. A model predicting EM revealed no significant interaction between cluster, PiB‐status and time (Figure 2). While a significant entorhinal tau x time effect was observed in a model predicting EM decline, cluster assignment did not modify this relationship. Results were similar when APOE4 status was included in the models.

**Conclusion:**

Favorable risk profiles (cluster 1 and 2) attenuated the effect of Aß, but not tau, on cognitive decline. These findings suggest that different risk profiles moderate pathology‐cognition relationships, and demonstrate that different factors may operate together in reducing risk. This highlights the role of groups of modifiable resilience factors in mitigating the effects of Aß deposition.